# Informed decision‐making: Statistical methodology for surrogacy evaluation and its role in licensing and reimbursement assessments

**DOI:** 10.1002/pst.2219

**Published:** 2022-07-12

**Authors:** Christopher J. Weir, Rod S. Taylor

**Affiliations:** ^1^ Edinburgh Clinical Trials Unit University of Edinburgh Edinburgh UK; ^2^ Institute of Health & Wellbeing University of Glasgow Glasgow UK

**Keywords:** licensing, reimbursement, surrogate outcome

## Abstract

The desire, by patients and society, for faster access to therapies has driven a long tradition of the use of surrogate endpoints in the evaluation of pharmaceuticals and, more recently, biologics and other innovative medical technologies. The consequent need for statistical validation of potential surrogate outcome measures is a prime example on the theme of statistical support for decision‐making in health technology assessment (HTA). Following the pioneering methodology based on hypothesis testing that Prentice presented in 1989, a host of further methods, both frequentist and Bayesian, have been developed to enable the value of a putative surrogate outcome to be determined. This rich methodological seam has generated practical methods for surrogate evaluation, the most recent of which are based on the principles of information theory and bring together ideas from the causal effects and causal association paradigms. Following our synopsis of statistical methods, we then consider how regulatory authorities (on licensing) and payer and HTA agencies (on reimbursement) use clinical trial evidence based on surrogate outcomes. We review existing HTA surrogate outcome evaluative frameworks. We conclude with recommendations for further steps: (1) prioritisation by regulators and payers of the application of formal surrogate outcome evaluative frameworks, (2) application of formal Bayesian decision‐analytic methods to support reimbursement decisions, and (3) greater utilization of conditional surrogate‐based licensing and reimbursement approvals, with subsequent reassessment of treatments in confirmatory trials based on final patient‐relevant outcomes.

## 1 INTRODUCTION

Andy Grieve's outstanding work in pharmaceutical statistics has spanned a broad spectrum of drug development activities, from toxicology studies, through later phase trials to manufacturing and pharmacoeconomics.^1^ A common thread has run through all of these areas regarding the valuable role of practical applications of Bayesian methodology. One distinctive feature, given the direct probabilistic inference that Bayesian approaches allow on parameters of interest, is to enable informed decision‐making during the development and deployment of pharmaceutical interventions.

Co‐author Weir first encountered Andy – or “Mr Bayes”, to use the moniker assigned by the clinical colleague who made the introduction – when on secondment with Pfizer Global Research & Development in the early 2000s. During the subsequent year of collaboration, the topic of statistical evaluation of candidate surrogate outcomes was one methodological area of focus. Since this field contains parallels with many of Andy's wider contributions to pharmaceutical statistics—in particular on the theme of statistical support for decision‐making in drug development—we select it for further attention in this reflective piece.

Driven by patients' and society's desire for faster access to therapies, and in parallel with the development of statistical methods for surrogate evaluation described below, there has been a long tradition of the use of surrogate endpoints in drug development. In addition to quicker market decisions (and return on R&D investment), surrogate endpoints also offer the healthcare industry the benefit of more efficient (smaller and quicker) and thus less costly clinical trial portfolios or, alternatively, the targeting of a broader population indication for a similar size of clinical trial.^2^

We consider the evolving role played by statistical methods for evaluating surrogate outcomes in the assessment of treatment efficacy by researchers, regulators and more recently in the decision‐making around reimbursement for the adoption of licensed medicines by healthcare systems. Themes include the contrasting approaches of causal inference and association‐based methods for surrogate evaluation; the potential use of Bayesian methodology; the role of surrogate evaluation frameworks in licensing to synthesize evidence on a surrogate; and the potential for decision‐analytic approaches to support reimbursement decision‐making. We conclude with recommendations on potentially fruitful areas for further exploration on this topic.

## 2 STATISTICAL METHODS FOR SURROGATE EVALUATION

### 2.1 Definitions and notation

To help orientate the reader, we adopt in this article the definitions for biomarker, clinical endpoint and surrogate endpoint established by The National Institutes of Health (NIH) Biomarkers Definitions Working Group^3^ (Box 1). We add the term intermediate endpoint which now has increasingly prominent usage in the schema of outcome measures, particularly in the field of health policy.^4^ Note that the final patient‐relevant outcome of interest is not necessarily clinical, for example in the case of health‐related quality of life.

BOX 1 **Definitions of biomarker, intermediate, surrogate endpoint and clinical endpoint**

*Biological marker (Biomarker)*: A characteristic that is objectively measured and evaluated as an indicator of normal biologic processes, pathogenic processes, or pharmacologic responses to a therapeutic intervention.

*Clinical endpoint*: A characteristic or variable that reflects how a patient feels, functions or survives.

*Intermediate endpoint*: A clinical endpoint such as measure of a function or of a symptom (disease‐free survival, angina frequency, exercise tolerance) but not the ultimate endpoint of the disease, such as survival or the rate of irreversible morbid events (stroke, myocardial infarction).^5^ Improvement in an intermediate endpoint due to treatment can be of value to the patient even if it does not lead to the improvement of morbidity or mortality.

*Surrogate endpoint*: A biomarker or intermediate endpoint intended to substitute for a clinical endpoint. A surrogate endpoint is expected to predict clinical benefit (efficacy/effectiveness) or harms.

In the notation that follows, we use S to denote the surrogate outcome, with S_ij_ indicating the value of the surrogate on participant *i* within trial *j*. Similarly T_ij_ refers to the value of the clinical or final patient‐relevant endpoint of interest on participant *i* in trial *j*. Z_ij_ will be used as an indicator of randomized treatment allocation for participant *i* within trial *j*, with a value of 0 for placebo or active comparator, and 1 for the intervention under investigation.

### 2.2 Statistical methods evaluating surrogate outcome validity: first steps

Since the pioneering step in surrogate evaluation introduced by Prentice in 1989,^6^ a plethora of extensions and alternative approaches has emerged. We would direct readers wishing a complete picture of the range of approaches available towards previously published reviews on the topic.^4^, ^7^

A non‐exhaustive list of methods (Table 1) indicates the considerable number and diversity of strategies developed, spanning the full range of types of surrogate and clinical outcomes. We now introduce a scheme for classifying methods and discuss in more detail a small number of approaches which have been influential in the overall development of thinking on surrogate evaluation. In particular we identify those which are novel or methodologically notable (such as the Prentice method and an early introduction of a Bayesian approach^8^); those which highlight the complementary roles of causal and associative methods (for example, principal stratification^9^ or direct and indirect effects^10^, ^11^); and those which have been widely adopted as tools for surrogate evaluation (such as meta‐analytic approaches,^12^ especially those grounded in information theory^13^).

**TABLE 1:** Statistical approaches to surrogate outcome evaluation.

References	Method	Type of surrogate	Type of clinical outcome
6	Prentice criteria (Prentice, 1989)	Time to event	Time to event
57	Proportion of treatment effect explained (Freedman et al, 1992)	Binary	Binary
58	Proportion of treatment effect explained (Lin et al, 1997)	Continuous	Time to event
59	Single trial: relative effect and adjusted association (Buyse and Molenberghs, 1998)	Continuous	Continuous
12	Multi‐trial: joint model for S and T (Buyse et al, 2000)	Continuous	Continuous
60	Multi‐trial: two stage model (Tibaldi et al, 2003)	Continuous	Continuous
8	Multi‐trial: Bayesian joint model (Daniels and Hughes, 1997)	All	All
61, 62, 63, 64	Multi‐trial: extension to time to event (Burzykowski et al, 2001; Renfro et al, 2012; Ghosh et al, 2012; Tibaldi et al, 2004)	Time to event	Time to event
65, 66, 67	Multi‐trial: extension to categorical outcomes (Molenberghs et al, 2001; Renard et al, 2002; Alonso et al, 2002)	Binary/ordinal/continuous	Binary/ordinal/continuous
21, 68, 69	Multi‐trial: repeated measures (Alonso et al, 2003; Alonso et al, 2004; Alonso et al, 2006)	Continuous	Continuous
70	Multi‐trial: repeated measures (Pryseley et al, 2010)	Continuous	Discrete
71	Multi‐trial: repeated measures (Renard et al, 2003)	Continuous	Time to event
23	Surrogate threshold effect (Burzykowski and Buyse, 2006)	All	All
72	Likelihood reduction factor (individual level surrogacy) (Alonso et al, 2004)	All	All
73, 74	Proportion of information gain (Qu and Case, 2007; Miao et al, 2012)	All	All
13	Multi‐trial: information theoretic approach (Alonso and Molenberghs, 2007)	Binary	Binary
75	Multi‐trial: information theoretic approach extension (Alonso and Molenberghs, 2008)	Time to event	Time to event
76	Multi‐trial: information theoretic approach extension (Pryseley et al, 2007)	Binary	Continuous
77, 78	Multi‐trial: information theoretic approach extension (Ensor and Weir, 2020; Ensor and Weir, 2021)	Binary/ordinal	Binary/ordinal
9, 19	Principal stratification, principal surrogate (Frangakis and Rubin, 2002; Li et al, 2011)	Binary	Binary
18	Principal stratification extension (Wolfson and Gilbert, 2010)	Continuous	Binary
79	Principal stratification extension (Conlon et al, 2014)	Continuous	Continuous
19, 25	Principal stratification extension – Bayesian(Li et al, 2010; Li et al, 2011)	Binary	Binary
80	Principal stratification extension – Bayesian (Zigler and Belin, 2012)	Continuous	Binary
81	Causal effect predictiveness (CEP) (Gilbert and Hudgens, 2008)	Discrete or continuous	Binary
82	CEP for time to event (Qin et al, 2008)	Continuous	Time to event
83	Average causal effect (Chen et al, 2007)	Discrete or continuous	Discrete or continuous
84	Distributional causal effect (Ju and Geng, 2010)	All	All
11	Direct and indirect effects via path analysis (Qu and Case, 2006)	All	All
85	Proportion of treatment effect explained – extension to joint modelling (Deslandes and Chevret, 2007)	Repeated measures Time to event (multistate)	Time to event Binary
86, 87	Structural equation modelling (Ghosh et al, 2010; Emsley et al, 2010)	All	All
88	Missing data perspective (Chen et al, 2003)	Continuous	Continuous
89	Missing data perspective (Chen et al, 2008)	All	All
90	Missing data perspective (Benda and Gerlinger, 2007)	Continuous	Binary
91	95% prediction model for true outcome (Baker et al, 2012)	Binary/time to event	Binary/time to event
92, 93	Joint modelling permitting multiple surrogates (Xu and Zeger, 2001; Lin et al, 2002)	Continuous	Time to event
94	Joint modelling incorporating repeated measures (Taylor and Wang, 2002)	Continuous	Time to event

### 2.3 Is a causal interpretation strictly necessary?

An enduring theme which has emerged in the evolution of statistical methodologies for surrogate evaluation is the question of causal inference. In this context, Joffe and Greene^14^ provided a helpful typology through which to classify methods for surrogate evaluation. They considered and contrasted two paradigms: first, that of causal effects (CE); and second, the concept of causal association (CA). Fundamentally, the causal association approach uses information on the relationship between the effect of treatment on the surrogate and the treatment effect on the clinical outcome to enable the surrogate effect to be used to predict treatment efficacy on the clinical outcome. In contrast, the causal effects paradigm uses the effect of treatment on the surrogate in combination with knowledge of the effect of the surrogate on the clinical outcome to draw inferences about the effect of treatment on the clinical outcome.

There has been substantial divergence in methodological applications to date, for example with causal effects approaches gaining much uptake in the field of vaccine development^15^ and causal association methods being widely implemented in oncology.^16^ Thoughts are now turning, however, towards commonalities between the CE and CA paradigms. For normally‐distributed S and T, Conlon and co‐authors provide helpful insights on the interdependencies of CE and CA.^17^ Adopting a structural model containing an unobserved confounder, they illustrate the impact of the identifiability assumptions required by CE methods on the quantification of surrogacy using CA approaches. In a reciprocal manner, they also highlight the impact that the assumptions made to support estimation under CA methods would have on CE parameters. We will refer to the causal effects and causal association classification when introducing the key methodological developments below and will subsequently comment on moves towards a more unified approach to surrogacy evaluation.

### 2.4 Key developments

#### 2.4.1 Prentice criteria

In a first investigation into formal statistical approaches for evaluating surrogate outcomes, Prentice^6^ stated that a surrogate should “yield unambiguous information about differential treatment effects on the true endpoint” and defined a confirmed surrogate endpoint as “a response variable for which a test of the null hypothesis of no relationship to the treatment groups under comparison is also a valid test of the corresponding null hypothesis based on the true endpoint.” This definition was then operationalized for *t*, the time to failure on a given clinical endpoint. In the notation below F(t) indicates, for clinical endpoint T, the distribution of censoring history and failure times up to time *t*. The key criterion is that surrogate S should capture all of the dependence of the clinical endpoint hazard rate λ_T_ on treatment group Z:

$$\lambda_{T}\;\left\{t|S\left(t\right),Z\right\}\equiv \lambda_{T}\;\left\{t|S\left(t\right)\right\}$$

Furthermore, S should predict T,

$$\lambda_{T}\;\left\{t|S\left(t\right)\right\}≢\lambda_{T}\;\left\{t\right\}$$

A further condition imposed is that the class of alternatives to the null hypothesis on S must alter the average true endpoint risk. In other words, any treatment effect observed on S must lead to a corresponding effect on the clinical endpoint T:

$$E\left[\lambda_{T}\left\{t|S\left(t\right)\right\}\;|\;Z;F\left(t\right)\right]≢E\left[\lambda_{T}\left\{t|S\left(t\right)\right\}\;|\;F\left(t\right)\right]$$

This ground‐breaking initial step in surrogacy evaluation prompted numerous attempts to build on this conceptual framework. It was subsequently classified as a CE method by Joffe and Greene.^14^

#### 2.4.2 Principal surrogacy

Frangakis and Rubin (F&R) introduced principal stratification as a method of modelling, for binary outcomes, the effect of a surrogate endpoint on a true endpoint.^9^ The key concept is to consider that, for example in a randomized parallel group clinical trial with two treatment groups, each participant would have two potential outcomes, one for each of the treatments in the trial, but only one of these can be observed, depending on the treatment group to which the participant was randomized.

While space limitations prevent a full exposition of the methodology, it will be useful to introduce the key terminology here. F&R maintained that causal effects should be the basis for surrogate endpoint evaluations, where the causal effect is a comparison between treatment groups of the potential outcomes on the same set of individuals.

The basic principal stratification for a surrogate partitions individuals so that within each stratum, all individuals have the same vector of potential outcomes for the two treatment groups. Building on this, the principal stratification partitions individuals by forming unions of the sets in the basic principal stratification. A principal effect compares outcomes across treatments within a principal stratum, and hence any principal effect is also a causal effect.

F&R then posited two requirements for surrogate validity. The first of these was causal necessity, which requires that an effect of treatment on T can only exist if treatment has also affected S. The second condition was statistical generalizability, which requires good predictive performance of S for T in a future study in which only S is observed.

On this basis F&R then established a new definition for surrogate validity: S is a principal surrogate when comparing the effect of treatments 0 and 1 on T if, for all fixed values s of the surrogate, the comparison of the principal strata of the ordered sets of potential outcomes {T_i_(0): S_i_(0) = S_i_(1) = s} and {T_i_(1): S_i_(0) = S_i_(1) = s} results in equality. A principal surrogate always meets the requirement of causal necessity.

Identifiability of counterfactual estimands under this methodology poses some challenges.^18^ Li et al (2011) addressed some practical aspects of implementation^19^ and further solutions are outlined in the *Bayesian* approaches section below. An extension from binary to time‐to‐event outcomes has been derived.^20^ Joffe and Greene^14^ consider principal surrogacy to belong within the CA class of methods.

From the contrasting Prentice and principal surrogacy approaches grounded in hypothesis testing and conceptual frameworks, a natural progression is then to consider methods which focus on the estimation of the strength of surrogacy.

#### 2.4.3 Meta‐analysis and information theory

It was rapidly recognized that a large quantity of data is required to estimate the value of a putative surrogate outcome. This led to several approaches making use of meta‐analysis techniques to allow all available data to be utilized. This in turn resulted in two levels of surrogacy performance being proposed, based on the coefficient of determination between the effects of treatment on the surrogate and clinical endpoint at the individual patient level (R^2^_indiv_) and overall in a clinical trial (R^2^_trial_).^12^

Initially, a joint mixed model, including random intercepts and random treatment effects across trials, was proposed for evaluating the effects of treatment on the surrogate and clinical outcomes.^12^ This proved to be computationally burdensome, and so the two‐stage fixed effects modelling approach presented by the same authors was a preferable alternative.

The original formulation for continuous outcomes was subsequently extended to a wide range of outcome types (Table 1). Here, the challenge was that in doing so the formulation of R^2^_indiv_ differed across contexts and for discrete outcomes in some cases had to be assessed at the latent variable level. The likelihood reduction factor (LRF)^21^ was introduced as a measure which could be applied consistently across different types of outcome.

Further theoretical support for a consistent framework across outcome types was sought using the information theoretic approach to quantifying uncertainty. Entropy quantifies the uncertainty in a discrete random variable, and entropy power extends this concept to continuous random variables.^22^ Using entropy power, Alonso and Molenberghs (2007)^13^ presented information theoretic surrogacy evaluation metrics for both the individual and trial levels. R^2^_h_ is based on entropy power (EP) and can be thought of as the proportion of uncertainty in T at the individual level removed by adjusting for S.

$$R_{h}^{2}=\frac{\mathrm{EP}\left(T\right)-\mathrm{EP}\left(T|S\right)}{\mathrm{EP}\left(T\right)}$$

An equivalent measure, R^2^_ht_, was derived for trial level surrogacy, this time based on the entropy power of the relationship between estimated treatment effects, α_i_ and β_i_, on each of S and T in trial *i*.

$$R_{\mathrm{ht}}^{2}=1-\frac{\mathrm{EP}\left(\beta_{i}|\alpha_{i}\right)}{\mathrm{EP}\left(\beta_{i}\right)}$$

Finally, a meta‐analytic R^2^_h_ was proposed to take account of between‐trial heterogeneity, given that *k* trials produce *k*_*q*_ possible R^2^_hi_:

$$R_{h}^{2}=\sum_{i=1}^{k_{q}}\tau_{i}R_{\mathrm{hi}}^{2}$$

where τ_i_ > 0 ∀ *i* and $\sum_{i=1}^{k_{q}}\tau_{i}$=1.

The choice of τ_i_ is drawn from an uncountable family of parameters; nevertheless, the LRF represents a consistent estimator of R^2^_h_ and supports unification across settings by providing a common interpretation regardless of the type of outcome being studied. The appeal of such a consistent treatment of surrogacy evaluation across settings has prompted extensions to other outcome types, including time to event, binary, ordinal, and repeated measures (Table 1). These meta‐analytic, information‐theoretic methods have been widely adopted as the preferred approach under the CA paradigm.

A natural question that follows is: at what level of such trial R^2^ or individual R^2^ measures should we conclude that a surrogate is valid? Some have proposed specific cut‐off values (for example, the surrogacy evaluation framework of the German Institute for Quality and Efficiency in Health Care [IQWiG], see Table 2), although context will surely also be important. Alternatively, the surrogate threshold effect (STE) has been created as a practical measure to define the minimum level of efficacy required on the surrogate to conclude that efficacy would also be present on the clinical endpoint.^23^

**TABLE 2:** International payers/HTA agencies & handling of surrogate endpoints: recommendations for specific statistical methods/considerations.

Country	Payer/agency	Statistical methods	Citation/web link(s)
United Kingdom	National Institute of Health & Care Excellence (NICE), 2013/9	“When the use of “final” clinical endpoints is not possible and “surrogate” data on other outcomes are used to infer the effect of treatment on mortality and health‐related quality of life, evidence in support of the surrogate‐to‐final endpoint outcome relationship must be provided together with an explanation of how the relationship is quantified for use in modelling. The usefulness of the surrogate endpoint for estimating QALYs will be greatest when there is strong evidence that it predicts health‐related quality of life and/or survival. In all cases, the uncertainty associated with the relationship between the endpoint and health‐related quality of life or survival should be explored and quantified. Multivariate meta‐analysis of summary data for combining treatment effects on correlated outcomes and evaluating surrogate endpoints: “When data on the final clinical outcome are not available or limited at the licensing stage, and therefore also for the HTA decision‐making process, a modelling framework is required to establish the strength of the surrogate relationship between the treatment effects on the surrogate and the final outcome and to predict the likely treatment effect on the final outcome for the new health technology. Multivariate meta‐analytic methods provide such a framework as they, by definition, take into account the correlation between the treatment effects on the surrogate and final outcomes as well as the uncertainty related to all parameters describing the surrogate relationship.” “Relying solely on patient‐level association is not sufficient when evaluating surrogate endpoints, in particular when individual‐level association has been evaluated based on data from a single trial (Fleming and DeMets 1996). A meta‐analytic approach based on data from more than one trial to establish the association between the treatment effects on the candidate surrogate endpoint and on the final outcome is more appropriate for evaluation of surrogate endpoints.”	https://www.nice.org.uk/process/pmg9/chapter/foreword http://nicedsu.org.uk/multivariate‐meta‐analysis‐tsd/
Germany	IQWiG, 2011	“There is no “best” method defined, however, correlation‐based validation is the ‘preferred’ method, in the sense it has been most widely used in evaluations. Another option discussed is the surrogate threshold effect (STE)” “A correlation of R ≥ 0.85; R^2^ ≥ 0.72 measured at the lower bound of the 95% percentage interval allows to conclude that the validation study represents a high reliable result. This interval R < 0.85; R^2^ < 0.72 to R > 0.7; R^2^ > 0.49 represents a medium reliable result between surrogate and patient relevant endpoint. If a validation study shows high reliable results with statistically low correlation (R ≤ 0.7; R^2^ ≤ 0.49) measured at the lower bound of the confidence interval then the surrogate is not considered as a valid endpoint.”	https://www.iqwig.de/download/a10‐05_executive_summary_v1‐1_surrogate_endpoints_in_oncology.pdf
Canada	CADTH, 2017	“Researchers should evaluate and justify the validity of any surrogate endpoints used for parameter estimation. Uncertainty in the association of the surrogate to the final clinical outcome should be reflected in the reference case probabilistic analysis. This uncertainty can also be explored through appropriate scenario analyses. The existence of multiple potential surrogates should be reflected in the analysis of uncertainty.”	https://www.cadth.ca/guidelines‐economic‐evaluation‐health‐technologies‐canada‐4th‐edition
Australia	PBAC 2008/2016	Transformation of a surrogate to a final outcome – suggested uncertainty analysis – use range of alternative plausible values and present as scenario analysis for economic evaluation Surrogate to Final Outcomes Working Group (STFOWG) report: Information requirements “For a meta‐regression of multiple randomised trials: the results for (1) the intercept and coefficient (and their 95% CI), (2) the coefficient of determination (R^2^ trial), (3) R^2^ individual (only if individual patient data are available for the trials), and (4) the surrogate threshold effect (STE) as determined by prediction bands.” “An assessment of the implications of the identified uncertainties relating to the PSM to TCO transformation for the structure, the input variables, the results and the sensitivity analyses of the modelled economic evaluation and for the presentation of the stepped economic evaluation.”	https://pbac.pbs.gov.au/information/printable‐version‐of‐guidelines.html https://www.pbs.gov.au/industry/useful‐resources/pbac‐technical‐working‐groups‐archive/surrogate‐to‐final‐outcomes‐working‐group‐report‐2008.pdf

Abbreviations: PSM, proposed surrogate measure; TCO, target clinical outcome.

#### 2.4.4 Bayesian approaches

As the meta‐analytic approaches discussed above evolved, a further CA strategy was proposed by Daniels and Hughes^8^ using a Bayesian random effects meta‐analysis. In essence, the model focuses on trial‐level effects as follows:

$$\theta_{i}\;|\;\gamma_{i}∼N\left(\alpha +{\beta \gamma }_{i};\tau^{2}\right)$$

where θ_i_ and γ_i_ are the treatment effects for trial *i* on T and S respectively. Minimally informative priors are placed on the fixed effects and the regression coefficients. An informative surrogate consistent with the Prentice criteria would have α = 0 and β ≠ 0; a perfect surrogate would also require τ^2^ = 0. At first glance it appears that use of the Bayesian approach may address some of the computational issues originally identified with the meta‐analytic approach involving the fitting of a joint mixed model with random intercepts and treatment effects; however, it has been noted that, as the number of trials in the meta‐analysis increases, inconsistent results are likely to arise due to the increasing number of nuisance parameters on which uninformative priors are being placed.^24^

Note that Bayesian approaches will also be readily applicable to most, if not all, of the methodologies outlined in Table 1. For example, Li et al (2010)^25^ implement the principal stratification approach using a Bayesian imputation technique to address problems of identifiability, and Elliott et al^26^ extend this further to incorporate missing data in the potentially observable final outcome under the scenarios of ignorable and non‐ignorable missingness. Bayesian inference also lends itself well to the regulatory and reimbursement decision‐making requirements discussed in Section 3.

### 2.5 Towards a unified approach

We conclude this methodology overview with an update on recent advances which have identified previously unrecognized connections between the causal effects and causal association principles. Alonso et al (2015)^27^ make a case for the use of expected causal effects, rather than individual causal effects, on the grounds that the expected causal effects are readily estimable and acceptable to regulatory authorities. They then explain that the results of a CA information theory‐based analysis generating an individual causal association and trial‐level surrogacy measure might be anticipated to be consistent with that of the individual causal effect. Further investigation will be needed to demonstrate whether this is the case across all types of surrogate and clinical endpoints. A subsequent development^28^ establishes a relationship between the individual causal association (CA) and the surrogate predictive function (CE) and implements a Monte Carlo approach to address identifiability problems. In the context of a binary surrogate and binary clinical outcome, Alonso et al (2018)^29^ offer a maximum entropy strategy from information theory to aid with identifiability in estimating the CE surrogate prediction function and the individual causal association (CA), and suggest that these measures may be used jointly to assess surrogacy. Thus, strands from the CE and CA paradigms have been combined in the quest for surrogacy evaluation. Alonso et al (2019)^30^ emphasize that this new measure of individual causal association does not substitute for the original R^2^_indiv_ which retains distinct value in its own right. Van der Elst et al (2021)^31^ go on to extend the original surrogate threshold effect metric from the CA paradigm to the new individual causal association in the CE context.

### 2.6 The next steps

It is clear from the above that the pharmaceutical statistician has a comprehensive range of methods at their disposal with which to quantify the efficacy of an intervention, based on evidence that includes data on one or more putative surrogate outcomes. We now go on to discuss the ways in which this evidence might be synthesized, alongside other factors such as quality, safety and cost‐effectiveness, to inform the decision‐making of regulatory authorities (on licensing) and payer and health technology assessment (HTA) agencies (on reimbursement).

## 3 USE OF SURROGATE ENDPOINTS IN HEALTHCARE POLICY

Access to drugs and other medical healthcare technologies across international health‐care systems depends on overcoming two evidentiary hurdles. First, regulatory bodies, such as the Food and Drug Administration (FDA) in United States, European Medicines Agency (EMA), and the Medicines and Healthcare products Regulatory Agency (MHRA) in United Kingdom, determine whether a healthcare technology should receive a license for entry to the market and be available for use in clinical practice. Such a license is typically based on three criteria: quality (are the raw materials, equipment, and the technical knowledge required to process, package, and distribute the therapy sufficient?), efficacy (does the therapy have demonstrable health benefit?) and safety (Is the therapy safe?). Second, subsequent to or in parallel with licensing, payers (often supported by HTA agencies) decide whether the therapy should be reimbursed, be that through a public funding or a private insurance system. Payer bodies, such as National Institute for Health and Care Excellence (NICE) in United Kingdom, Institute for Quality and Efficiency in Health Care (IQWiG) in Germany, or Centre for Medicare and Medicaid (CMS) in United States, raise two additional evidentiary criteria: comparative effectiveness (does the therapy provide sufficient health benefit over the longer term compared to standard of care?) and cost‐effectiveness (does the benefit relative to the cost of therapy provide good value for money?).^32^ Surrogate endpoints can play a key role in both the licensing and reimbursement settings.^4^, ^33^

### 3.1 Licensing

Acceptance based on surrogate outcomes was initially limited to specific licensing circumstances. In response to the AIDS epidemic of the 1980s, the FDA introduced its “accelerated approval” regulation in 1992 to approve new drugs or biologicals intended for treatment of “serious illnesses” that offer meaningful therapeutic benefit compared with existing treatment on the basis of a documented treatment effect on a surrogate or non‐ultimate endpoint.^34^ Accelerated approval focuses on unmet medical need and can include chronically debilitating conditions, where the number of patients in trials is likely to be small and there is no existing licensed therapy, or orphan medicines, used against rare diseases, often also without existing treatments. Although initially developed for accelerated approval for life threatening diseases without treatment options, agencies such as the FDA and EMA have steadily expanded use of surrogates far beyond this original intent. In 2018, 73% of FDA licensed drugs (43/59) received expedited approval and many of these were for conditions such as gout and hypertension that were neither life‐threatening nor lacking in existing treatments.^35^ A review of FDA cancer drug approvals between 1992 and 2019 found 194 drug authorizations for 132 drugs that were based on surrogate endpoints.^36^ Only half (89, 46%) of these approvals were based on accelerated licensing pathways, the remainder being under the regular licensing process. Furthermore, pivotal trials using surrogates as their primary endpoint formed the exclusive basis of FDA approvals for 91 of 206 (44%) indications for novel therapeutic agents between 2005 and 2012.^37^

**FIGURE 1:**
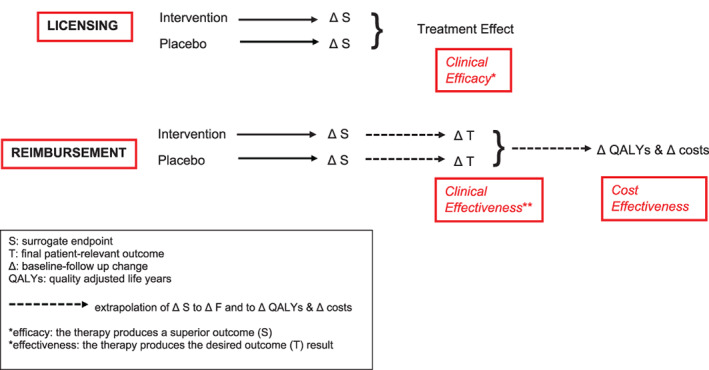
Comparison of role of surrogate endpoints in licensing and reimbursement.

The potential risk of using surrogate endpoints in the regulatory setting has long been highlighted,^38^ based on cases where drugs licensed on the basis of surrogates later turned out to be harmful and increase overall mortality. As the authors note, these effects can be the results of unintended effects of surrogate endpoints in the pathway of the specific disease being treated and action of the therapy (Figure 1). There may exist (1) non‐causal associations: a surrogate endpoint that is thought to be causal might simply be associated; (2) multiple causal pathways: a surrogate endpoint may lie within just one of several causal pathways of disease, thereby any changes in the surrogate may have an uncertain, and potentially negligible, effect on the desired patient outcome; (3) unintended outcomes: the therapy may exert effects outside the disease process that can have unmeasured harm or unmeasured benefit on patient outcomes. Even outcomes which appear biologically plausible as surrogates, such as nonfatal myocardial infarction as a surrogate for all‐cause or cardiovascular mortality, can fail to achieve acceptable levels of surrogacy.^39^

While it cannot completely prevent the unintended negative final health outcome consequences of using surrogates, it has been proposed that this risk can be reduced by restricting licensing claims to new therapies for which there is an appropriately validated surrogate endpoint.^35^ However, use of statistically validated surrogates does not appear to have been consistently implemented in the regulatory setting. In the above survey of FDA cancer drugs,^36^ the strength of association between the surrogate endpoint (such as progression free survival or tumour response) and the final outcome of all‐cause mortality was often weak or absent. A total of 61% of approvals had no documented correlation between the surrogate and mortality, 16% had poor correlation (r ≤ 0.7), 2% had medium correlation (0.70 < r < 0.85), 5% had high correlation (r ≥ 0.85), and 17% had varied levels of correlation across multiple validation studies. The FDA's current table of surrogate endpoints is based on either surrogate endpoints accepted by FDA in their previous approvals, or those the FDA consider may be acceptable for future approval application.^40^ However, rather paradoxically, the surrogate endpoints listed in the FDA table do not necessarily have evidence of their formal statistical validation.^41^ A study of EMA authorizations issued between 2011 and 2018 for products assessed by expedited pathways, found the majority of approvals were based on pivotal trials that reported non‐validated surrogate endpoints.^42^

Although we have focused on the role and challenges of surrogate endpoints in licensing for drug and biologic therapies, it is important to acknowledge that these considerations equally apply to other therapies, such as medical devices.^43^

### 3.2 Payers/reimbursement/HTA

As outlined above, reimbursement decisions by the payer and HTA agencies (hereafter referred to as payer for brevity), such as NICE in the United Kingdom, are typically made based on estimates of clinical effectiveness and cost‐effectiveness of new therapies relative to current standard of care. When only the surrogate endpoint from a clinical trial programme (and the regulatory process) is available to assess the cost‐effectiveness of medical technology, payers are therefore required to extrapolate the long‐term estimates of clinical effectiveness that can include many parameters including costs and utilities, to populate a health economic decision model.^44^

Although for a given new therapy, the same trial evidence of surrogacy is likely to be used in reimbursement as licensing, there is an important fundamental difference in the decision process between settings (Figure 1). Licensing approval of a therapy is likely to follow from demonstration of statistical superiority (typically against placebo in the phase 3 trial setting) on a validated surrogate endpoint, that is, “clinical efficacy” (the therapy produces a superior outcome result). However, in the payer setting, the magnitude of the predicted treatment effect on the final patient relevant outcome based on the observed treatment effect on the surrogate endpoint is also key. The relative magnitude of treatment effect on the surrogate needs to be sufficient to predict a meaningful long‐term improvement in the clinical outcome, (e.g., achievement of surrogate threshold effect – see Table 2, which summarizes the statistical considerations for surrogate evaluation recommended by international payer and HTA agencies) that is, “clinical effectiveness” (the therapy produces the desired outcome[s] result). If this can be shown, the payer then needs to judge that the therapy is “cost effective.” In the case of NICE this requires the extrapolation of final outcome into quality adjusted life years (QALYs), with demonstration that the incremental cost per QALY gained with therapy is acceptable.^45^ A key aspect of the reimbursement decision by payers is therefore to assess the magnitude of therapeutic benefit of a medical technology (relative to additional cost over current standard care). In this context, payers face the further challenge that trials of a surrogate endpoint can lead to substantial overestimation of the treatment effects compared to clinical outcomes. In their review of 324 cardiovascular trials, Ridker and Torres found that trials with primary endpoints that were surrogates were more likely to report a positive treatment effect (77 of 115 trials; 67%) than trials that reported final patient‐relevant primary outcomes (113 of 209 trials; 54%, *P* = 0.02).^46^ Many of these trials informed licensing decisions. A meta‐epidemiological study involving 185 randomized controlled trials reported in six high‐impact general medical journals that used surrogate endpoints or patient‐relevant outcomes compared the treatment effects from trials that used surrogates and those that used final outcomes.^47^ This analysis found that trials using surrogate endpoints were more likely to report positive treatment effects as trials using final outcomes (52 of 84 trials [62%] vs. 37 of 101 trials [37%], *P* < 0.01). Furthermore, trials that used surrogates found treatment effects that were, on average, 28% to 48% larger than trials that used corresponding clinical outcomes. So, in addition to the requirement for surrogate validity (i.e., treatment effect on surrogate has a strong association with clinical outcome effect), in order to assess clinical and cost effectiveness payers must also know the magnitude of the surrogate‐clinical outcome association (and the uncertainty around this) in order to model reliably the long‐term clinical and cost‐effectiveness of a therapy.^4^

To assist payers in appropriate use of surrogate endpoints in their decision‐making, two evaluative frameworks have been developed. The first was originally proposed by Taylor and Elston,^48^ based on the JAMA checklist for clinical trials,^49^ and updated by Ciani in 2017.^4^ We summarize the evaluative framework in Table 3. Here, level 3 evidence for a surrogate is based on biological plausibility alone, whereas evidence is considered to be level 2 when a strong association exists between the surrogate and the clinical endpoint across cohorts or at the level of the individual patient. The highest level of evidence (level 1) requires evidence that technologies that improve the surrogate also improve the clinical outcome across many randomized controlled trials. In 2008, Lassere proposed a multidimensional hierarchical evidence schema for evaluating the status of biomarkers as surrogate endpoints ‐ Biomarker‐Surrogacy Evaluation Schema (BSES).^50^ BSES extends beyond the Ciani framework to include the dimensions of biological, epidemiological, statistical, clinical trial and risk–benefit evidence of the biomarker clinical endpoint relationships. BSES systematically evaluates and ranks the surrogacy status of biomarkers and surrogate endpoints using defined levels of evidence. The schema incorporates three independent domains: study design (the highest score being ≥3 RCTs in the appropriate drug classes assessing the relationship between surrogate and final outcome), target outcome (the highest score being for a clinical outcome of mortality), and statistical evaluation (the highest score occurring where an intervention change in the surrogate strongly predicts change in the clinical outcome based on a statistical measure such as R^2^_trial_, R^2^_indiv_ or STE). Each domain ranks items from 0 to 5. An additional category called “penalties”, due to lack of evidence, evidence to the contrary or evidence of harm, incorporates additional considerations of biological plausibility, benefit–risk and generalizability. The total score (0–15) determines the level of evidence, with Level 1 the strongest and Level 5 the weakest. To be designated a Level 1 or 2 surrogate, a biomarker or intermediate outcome must also meet the rank of at least 3 within the study design, target outcome and statistical strength domains, and there must be no RCT evidence that use of the biomarker caused patient harm.

**TABLE 3:** Hierarchy of evidence for surrogate endpoints.

	Definition	Source of evidence	Statistical metrics
Level 3	Biological plausibility	Clinical data and understanding of disease (surrogate endpoints on final patient relevant outcome disease pathway)	Not applicable
Level 2	Observational association	Epidemiological studies of relationship between surrogate endpoint & final patient relevant outcome	Correlation between surrogate endpoint and final patient relevant outcome
Level 1	Interventional/treatment effect association	Randomised controlled trial(s)^a^ with treatment change in surrogate endpoint and final patient relevant outcome^b^	Trial level R^2^/Spearman's correlation Surrogate threshold effect (STE)

*Note*: Adapted from Ciani et al, 2017^4^; Taylor & Elston, 2009^48^; Bucher et al, 1999.^49^

^a^ Individual participant or trial level meta‐analysis of multiple randomised controlled trial or single large randomised controlled trial with surrogate‐final outcome association assessed by trial site.

^b^ Evidence should be randomised controlled trials from same disease indication, line of therapy, class of treatment/intervention and comparator therapy that surrogate endpoint is applied. If extrapolating from different disease indication, line of therapy, class of treatment/intervention and comparator therapy then evidence may not qualify as level 1.

Despite a clear preference for patient‐relevant final endpoints and therefore caution around the use of surrogates, there has been no universal uptake of these evaluative frameworks by international payers.^4^ Furthermore, few payers have specific methodological guidance around the assessment of medical technologies based on surrogate outcomes. Grigore et al reported that across 73 HTA agencies, only 29 (40%) had methods guidance that specifically referred to surrogate endpoints. Furthermore, only a very small number of these agencies (NICE in United Kingdom, Australia Pharmaceutical Benefits Advisory Committee (PBAC), Canadian Agency for Drugs and Technologies in Health (CADTH), and German Institute for Quality and Efficiency in Health Care (IQWiG)) had developed more detailed analytic methods and prescriptive criteria for the acceptance of surrogate endpoints, including proposed statistical methods for surrogate validation^43^ (see Table 2). While these agencies each acknowledge the lack of methodological consensus including statistical analytic approaches around the validation of surrogates, there is clear emphasis on their preference for multiple randomized trial data supporting the link between surrogate and clinical endpoints (supported by meta‐analysis/meta‐regression). NICE methods, informed by their technical advisory center (Decision Support Unit, DSU) report,^51^ recommend the use of Bayesian multivariate meta‐analytic methods to take into account the correlation between the treatment effects on the surrogate and clinical outcomes. However, only one agency (IQWiG) discusses acceptable cut‐off numerical values of the level of association between surrogate and final outcome and indicates that the lower bound of the 95% percentage interval must include a correlation (r) ≥0.85 or regression‐based model R^2^ ≥ 0.72 to indicate high validity. NICE have recently updated their methods, processes, and topic selection for health technology evaluation.^52^ As part of this update, NICE commissioned a number of technical support papers that included considerations on the handling of surrogate outcomes that include the following specific recommendations: “In all cases, the uncertainty associated with the relationship between the surrogate endpoint and the final outcome should be quantified and presented. It should also be incorporated through probabilistic sensitivity analysis and can be further explored in scenario analysis.” and “Extrapolation of a surrogate to final to a different population or technology of a different class or with a different mechanism of action needs thorough justification.”^53^

## 4 THE WAY FORWARD?

In the light of the technical developments and healthcare policy considerations outlined above, we make the following observations and suggestions for areas that merit prioritized attention going forward:

1. With the increasing patient and societal pressure for faster access to therapies (as highlighted by the development of vaccines and therapies in response to the COVID‐19 pandemic), there is likely to be increased future pressure for more efficient clinical trial design, including use of surrogate endpoints, to inform licensing and reimbursement of new therapies.
2. Avoid the use of accelerated/expedited access approaches based on non‐validated surrogates, and limit their use (based on validated surrogate endpoints) to those circumstances where such expedited evaluation is necessary and warranted, for example, diseases/conditions that severely impair and have high unmet treatment needs. The UK Early Access to Medicines Scheme (EAMS)^54^ is one such framework which targets use through defined eligibility criteria.
3. Given growing consensus regarding statistical methods for the evaluation and validation of surrogate endpoints, increase the regulatory pressure to apply surrogate evaluation frameworks with statistical validation at their heart as part of licensing and reimbursement decision‐making, to focus on therapies with validated surrogate endpoints.
4. Consider quantitative decision analytic frameworks to assist reimbursement decision‐making that include a transparent quantification of the predicted effect on the clinical outcome and related health outcomes (such as QALYs) of an observed trial‐based change in the surrogate. Such models could incorporate surrogate outcome evidence informed by Bayesian multivariate meta‐analysis.^51^
5. Increase application of conditional models of licensing and reimbursement where therapies are reassessed using confirmatory trials based on final patient relevant outcomes (and withdrawn if these fail to show predicted benefit and economic outcomes). Conditional assurance may be used to estimate the probability of success in a confirmatory trial where initial approval has been based on a surrogate outcome.^55^
6. Improved reporting of randomized trials using surrogate endpoints is needed including the SPIRIT (Standard Protocol Items: Recommendations for Interventional Trials) 2013 and CONSORT (Consolidated Standards of Reporting Trials) 2010 statements. The ongoing work on extensions to these documents (SPIRIT‐SURROGATE and CONSORT‐SURROGATE) provides a timely opportunity to engage researchers, regulators and other key stakeholders in the challenges of the use of surrogates in clinical trials.^56^

## 5 CONCLUDING REMARKS

Since the initial forays into surrogate endpoint evaluation over 30 years ago, development of multiple methodologies has rapidly taken place and continues apace. As understanding of how to interpret the outputs generated by these methods grows, we must now move to incorporate them formally as a quantitative input to licensing and reimbursement decision‐making. A Bayesian approach to this would provide a natural framework through which to take full account of the uncertainties inherent where the accumulated evidence incorporates surrogate outcome data.

## CONFLICT OF INTEREST

The authors have no interests to declare.

## Data Availability

Data sharing is not applicable to this article as no new data were created or analyzed in this study.
